# Localized crystallization in shear bands of a metallic glass

**DOI:** 10.1038/srep19358

**Published:** 2016-01-13

**Authors:** Zhijie Yan, Kaikai Song, Yong Hu, Fuping Dai, Zhibing Chu, Jürgen Eckert

**Affiliations:** 1 School of Materials Science and Engineering, Taiyuan University of Science and Technology, Taiyuan, 030024, P. R. China; 2 IFW Dresden, Institut für Komplexe Materialien, Helmholtzstraße 20, D-01069 Dresden, Germany; 3 School of Mechanical, Electrical & Information Engineering, Shandong University (Weihai), Weihai 264209, P.R. China; 4 Northwestern Polytechnical University, Xi’an 710072, P.R. China; 5 Erich Schmid Institute of Materials Science, Austrian Academy of Sciences, Jahnstraße 12, A-8700 Leoben, Austria; 6 Department of Materials Physics, Montanuniversität Leoben, Jahnstraße 12, A-8700 Leoben, Austria

## Abstract

Stress-induced viscous flow is the characteristic of atomic movements during plastic deformation of metallic glasses in the absence of substantial temperature increase, which suggests that stress state plays an important role in mechanically induced crystallization in a metallic glass. However, it is poorly understood. Here, we report on the stress-induced localized crystallization in individual shear bands of Zr_60_Al_15_Ni_25_ metallic glass subjected to cold rolling. We find that crystallization in individual shear bands preferentially occurs in the regions neighboring the amorphous matrix, where the materials are subjected to compressive stresses demonstrated by our finite element simulations. Our results provide direct evidence that the mechanically induced crystallization kinetics is closely related with the stress state. The crystallization kinetics under compressive and tensile stresses are interpreted within the frameworks of potential energy landscape and classical nucleation theory, which reduces the role of stress state in mechanically induced crystallization in a metallic glass.

Metallic glasses are of interests due to their significant academic and practical values^1^,^2^,^3^. However, due to their thermodynamically metastable nature, metallic glasses tend to transform into more stable phases upon heating^4^. Furthermore, it has been found that plastic deformation induces crystallization in metallic glasses^5^,^6^,^7^,^8^,^9^. Though extensive efforts have been devoted to understanding the mechanism of mechanically induced crystallization, one main controversy on this issue is whether the crystallization is caused by the shear-induced viscous flow or the local heating increase because there is a considerable debate about the temperature increase during severe plastic deformation, which is estimated to be from a few Kelvin up to thousands of Kelvin^10^,^11^,^12^. The local temperature effect can not explain the phenomenon that nanocrystallization only occurs on the compressive side in a bent Pd-based metallic glass^13^. This suggests that viscous flow is a crucial driving force for the redistribution of atoms during plastic deformation, which has been demonstrated in a Zr-based bulk metallic glass due to cold rolling^14^. The viscous flow is expected to result in the variation of stress along the width of a shear band due to the confining effect of the surrounding amorphous matrix. This invites a question whether and how the resultant stress sates affect the crystallization kinetics in individual shear bands. However, it is poorly understood. Here, to reduce the role of stress state in mechanically induced crystallization, we investigate the correlation between the stress distributions and the crystallization kinetics in individual shear bands of Zr_60_Al_15_Ni_25_ bulk metallic glass due to cold rolling.

Rolling, a representative severe plastic deformation method, was employed in the present work. The advantages of cold rolling are that the uncertainties in strain rate and temperature increase can be safely excluded. Furthermore, the influence of contamination on the phase transformation during rolling is minimized with respect to other severe plastic deformation methods (e.g., ball milling). However, it is nearly impossible to directly investigate the stress distributions in individual shear bands (with a width of up to a few micrometers^5^,^6^,^7^,^8^,^9^) within an experimentally accessible time window. Alternatively, finite element simulation provides a powerful tool for studying the stress distributions in micron ranges during rolling of materials^15^,^16^. In the present work, finite element simulation was employed to investigate the stress distributions in individual shear bands during cold rolling of Zr_60_Al_15_Ni_25_ bulk metallic glass.

## Results

**Figure 1:**
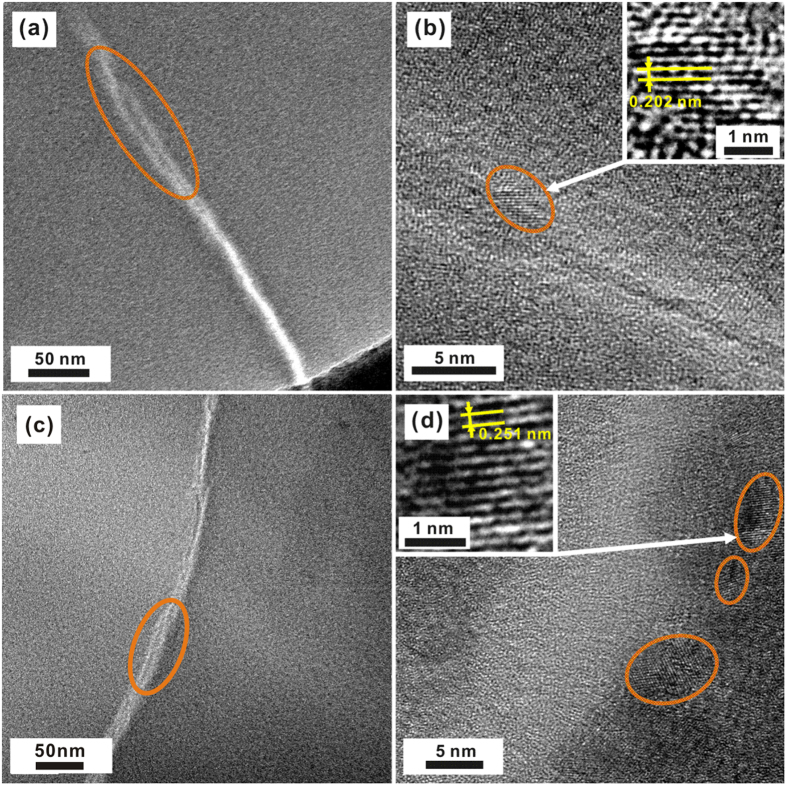
(**a**) HRTEM image of a rolled sample with the deformation degree of 40%. (**b**) Magnified image of the local region enclosed by the ellipse in (**a)**. (**c**) HRTEM image of a sample with the deformation degree of 80%. (**d**) Magnified image of the enclosed region by the ellipse in image (**c**), and the inset is a higher magnification image of a nanocrystal, showing the occurrence of nanocrystallization in the regions neighboring the amorphous matrix.

The X-ray diffraction (XRD) pattern and transmission electron microscopy (TEM) image (see Supplementary Information, Fig. S1) verify the amorphicity of as-cast Zr_60_Al_15_Ni_25_ bulk specimens. Figure 1a presents a high-resolution transmission electron microscopy (HRTEM) image of a rolled sample with the deformation degree *ε* of 40% (see its definition in Method section). A bright contrast region, being thinner than the surrounding undeformed amorphous matrix due to its lower resistance to chemical attack during electrolytic thinning, is seen in Fig. 1a and termed a shear band. Figure 1b is a magnified HRTEM image of the region enclosed by the ellipse in Fig. 1a, showing the precipitation of nanocrystals with a lattice spacing of 0.202 nm (the inset). Figure 1c presents a HRTEM image of a rolled sample with ε of 80%, and a magnified image of the enclosed region is shown in Fig. 1d, indicating the precipitation of nanocrystals (see the enclosed regions by ellipses). A higher magnification image of a nanocrystal (the inset in Fig. 1d) indicates that a lattice spacing of the crystalline fringe is 0.251 nm. Based on the HRTEM images, it is clearly seen that nanocrystallization in individual shear bands preferentially occurs in the regions neighboring the amorphous matrix rather than in the centre regions, which is also observed in individual shear bands of Cu-based bulk metallic glasses due to uniaxial compression^17^. The energy dispersive spectrometry (EDS) patterns (see Supplementary Information, Fig. S2) indicate that the average chemical composition of amorphous matrix is Zr_59.03_Al_16.41_Ni_24.33_, being consistent with the nominal composition of Zr_60_Al_15_Ni_25_. The EDS patterns (see Supplementary Information, Fig. S3) suggest significant redistribution of atoms in individual shear bands. The electron energy loss spectroscopy (EELS) patterns (see Supplementary Information, Fig. S4) indicate that the oxygen content in a shear band is negligible, which implies that the HRTEM samples are prevented from oxidation during thinning and the influence of oxygen on the crystallization kinetics is excluded. The thermal profiles around shear bands during inhomogeneous deformation of metallic glasses are difficult to be probed by experiment within the nanosecond time window of shear banding. The calculated thermal profile in a shear band shows that the highest local temperature increase is located at the shear plane, which results in occurrence of a hot zone behind the moving shear band front^18^. This means that, if the local heating effect works, heating-induced crystallization in a shear band should preferentially occur on the shear plane (the central region of the shear band) rather than on the region neighboring the undeformed matrix. However, our observed localized nanocrystallization in individual shear bands (Fig. 1) is a different scenario, which suggests that the localized nanocrystallization is induced rather by viscous flow than by local heating.

**Figure 2:**
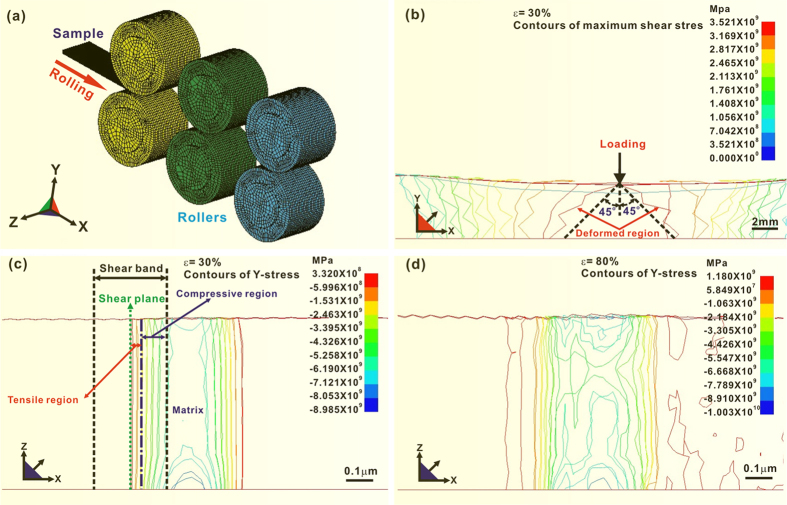
(**a**) Simulation model of rolling. (**b**) Contours of maximum shear stresses on the longitudinal X-Y section of a rolled sample with the deformation degree of 30%; Contours of Y-stress in a local deformed region, including 2 shear bands and the matrix between them, under the deformation degrees of 30% (**b**) and 80% (**c)**, indicating that a shear band is composed of the tensile and compressive regions.

The simulation model is shown in Fig. 2a (more details about simulation see Methods section). Figure 2b presents the contours of maximum shear stresses distributed on the whole longitudinal X-Y cross-section of a rolled sample with *ε* of 30%. The shear yield stress of Zr_60_Al_15_Ni_25_ bulk metallic glass is approximately 1.0 × 10^9 ^Pa (see Supplementary Information, Fig. S5). It is obvious that the maximum shear stresses in some localized regions exceed the shear yield stress, where shear-induced plastic deformation occurs according to Tresca yield criterion. The deformed regions (indicated by the arrows) are located at the planes having a ~ 45 degree to the loading direction due to rolling. These localized plastic deformed regions termed shear bands are substantiated by the HRTEM images (Fig. 1). To systematically investigate the stress distribution in a shear band, the stress contours of a local deformed region, including 2 shear bands and the matrix between them, are presented in Fig. 2c. To highlight the stress distribution in a shear band, the shear bands are presented in halves. During cold rolling, the materials in individual shear bands are loaded by complicated stresses rather than uniaxial stresses. Hence, a component stress along a defined direction (e.g., Y axis, see Fig. 2a) was employed to evaluate the stress distribution in individual shear bands. Figure 2c is the contours of Y-stress (i.e., the stress distribution along the direction of Y axis) within a local area under *ε* of 30%. The plus and minus values in the contours stand for the tensile and compressive stresses, respectively. In Fig. 2c, it is shown that the materials near the shear plane in a shear band are subjected to tensile stresses, being in a dilated state; and the stresses between the dilated region and the undeformed matrix show minus values, indicating that the materials neighboring the undeformed matrix are subjected to compressive stresses. In this sense, a shear band is suggested to be composed of two regions, i.e., the tensile center region and the compressive border region neighboring the amorphous matrix (Fig. 2c). Figure 2d presents the contours of Y-stress of the shear bands (shown in Fig. 2c) under the deformation of ε = 80%, which indicates that the compressive and tensile regions of shear bands develop due to further deformation. This indicates that the shear bands propagate with the increasing of deformation degree. The maximum compressive stresses under *ε* of 30% and 80% are −9.89 × 10^9 ^Pa and −1.00 × 10^10 ^Pa (Fig. 2c,d), respectively. It is worth explaining that the simulated stresses along the width of a shear band may deviate from the actual values since the simulation model has a slight discrepancy from the width of a shear band. However, the qualitative stress distributions in individual shear bands indicated by the simulation results are reasonable and convincing.

## Discussion

As a metallic glass is subjected to deformation at the temperature far below its glass transition temperature at the level of the shear yield stress, inhomogeneous plastic deformation occurs with the characteristic of formation of localized shear bands (Fig. 1). More excess free volume is introduced in shear bands during their nucleation and propogation^19^,^20^, which is attributed to the tensile effect indicated by the simulation results (Fig. 2c,d). As a result, viscosity substantially decreases in shear bands, which is in a similar range measured in the supercooled liquid region^21^. Consequently, the more liquid-like atomic configuration results in collective motion of atoms, involving of tens of atoms termed shear transformation zones (STZs)^22^,^23^,^24^. The accumulating of excess free volume results in viscous flow. However, due to the confining of surrounding undeformed amorphous matrix, the materials neighbouring the amorphous matrix are loaded by compressive stresses (Fig. 2c,d).

**Figure 3:**
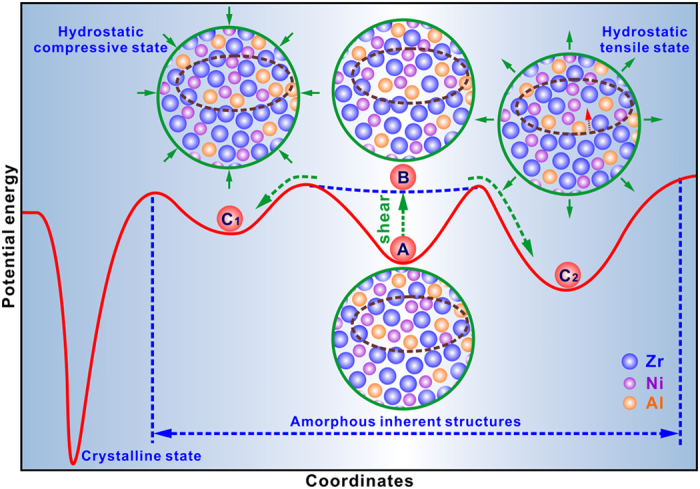
Schematic illustration of a potential energy landscape, including the amorphous inherent structures and the crystalline state. The potential energy minimum (PEM) *A* of a cluster flattens and finally disappears due to shear, resulting in a mechanically unstable configuration of *B*, in which STZs (exemplified by a circled region) are triggered. The configuration *B* undergoes structural rearrangements to the alternative PEM *C*1 and PEM *C*2 under hydrostatic compressive and tensile effects, respectively.

Within the framework of potential energy landscape (PEL) theory^25^, a potential energy function is used to model the energetic landscape of a system, which comprises a population of inherent structures associated with local minima being the stable states of metallic glasses. These inherent structures are separated by saddle points that constitute the barriers for configurational hopping among different amorphous states. Figure 3 presents a schematic illustration of a proposed PEL, which includes the amorphous inherent structures and a crystalline state. STZ hypothesis is applicable for different glasses based on the concept of PEL^26^, which indicates that STZs are the structural units in the stress-induced viscous flow. As an example, a cluster is sampled in a potential energy minimum (PEM) *A*. When the strain increases due to cold rolling, the PEM *A* flattens and finally is configured in a configuration *B*, in which STZs (exemplified by a circled region) are triggered. The configuration *B* becomes mechanically unstable and undergoes structural rearrangement to an alternative PEM. It’s reasonably considered that nano-sized STZs are under hydrostatic stress states. Under a hydrostatic compressive effect, the cluster tends to be configured in a PEM (e.g., *C*1) without release of elastic energy. Under the compressive state, the atoms in the STZ become jammed, which enhances the atomic redistribution to form an orderly configuration, as substantiated by the molecular dynamics simulations^27^. On the contrary, the cluster prefers to be configured into another PEM (e.g. *C*2) by releasing the elastic energy under a hydrostatic tensile effect^28^. Under this situation, more excess free volume is introduced in some triggered STZs (indicated by arrows in the PEM *C*2). From Fig. 3, it is clear that the nucleation barrier for nucleation of the cluster sampled in the PEM *C*1is considerably lower than that in the PEM *C*2.

For an amorphous-to-crystalline phase transformation, the thermodynamic energy barrier (∆G^*^) for homogeneous nucleation of a nucleus with a critical radius of *r* under a hydrostatic pressure can be expressed as^17^


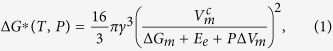


where *T* is the temperature, *P* is the hydrostatic pressure, *∆G*_*m*_ is the molar free energy change for the amorphous-to-crystalline phase transformation, *γ* is the interfacial free energy between the crystalline and the amorphous phases, 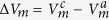 is the volume change due to the formation of a crystalline phase (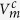 and 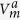 are the molar volumes of the crystalline and the amorphous phases, respectively), and *E*_e_ is the elastic strain energy induced by a volume change due to phase transformation, expressed as 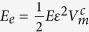, where *E* is Young’s modulus, and 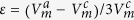.

Based on the fringe spacing (Fig. 1b,d) and the EDS pattern (see Supplementary Information, Fig. S6) of the precipitated phase, the crystalline crystals are identified to be a simple tetragonal Zr_2_Ni phase with *a* = *b* = 0.649 nm and *c* = 0.528 nm^29^. It is concluded that tetragonal Zr_2_Ni crystals nucleate in shear bands. There are 2 Zr and 1 Ni atoms in a unit cell of Zr_2_Ni. The ratio of molar volume amorphous phase to crystalline phase (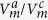) is assumed to be approximately 1.16 30. The values of 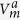, 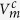 and *E*_*e*_ are calculated and shown in Table 1. As a crystalline nucleus forms in an amorphous phase, an interface between the crystalline and amorphous phase appears simultaneously. The interfacial free energy *γ* is a critical factor influencing crystallization kinetics. Turnbull has found that a ratio *α* of the interfacial free energy to the melting enthalpy (i.e., 
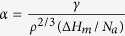
, where 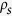
is atoms per unit volume (atoms/m^3^), 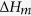 is the melting enthalpy (J/mol), and 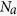 is Avogadro’s number) is nearly a constant of 0.45 for most metals^31^. 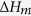 of Zr_60_Al_15_Ni_25_ alloy is approximately 14.3 kJ/mol^32^. Then, the interfacial free energy (*γ*) between the crystalline Zr_2_Ni phase and the amorphous Zr_60_Al_15_Ni_25_ phase is calculated to be approximately 0.1 J/m^2^. The value of ∆*G*_*m*_ is needed to calculate ∆G^*^(*T*, *P*). By setting *P* = 0, Eq. (1) is expressed as 
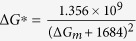
(kJ/mol). The activation energy for crystallization (*E*_*c*_) in a metallic glass can be expressed as
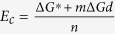
, where and ∆*G*_*d*_ is the activation free energy for the transfer of flow units (kinetic barrier) that is approximately 120 kJ/mol for Zr-based metallic glasses^33^. The values of *m* and *n* are 3 and 4, respectively, for the exothermic nucleation of spherical nuclei during heating^34^. *E*_*c*_ of Zr_60_Al_15_Ni_25_ metallic glass is determined to be approximately 345 kJ/mol by Kissinger analysis^35^. The value of *∆G*_*m*_ is calculated according to expressions of ∆G^*^ and *E*_*c*_ and shown in Table 1.

Based on the values of parameters shown in Table 1, the relation between ∆G^*^and *P* for the amorphous-to- crystalline phase transformation in Zr_60_Al_15_Ni_25_ metallic glass due to rolling can be expressed as


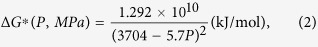


**Figure 4:**
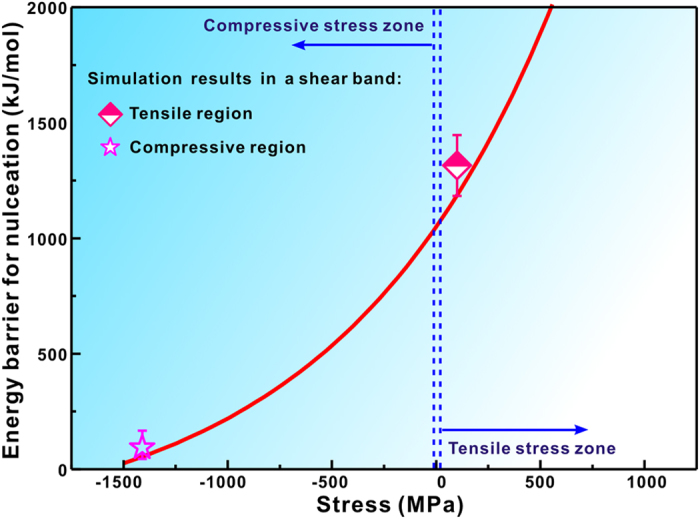
Relation between the energy barrier for nucleation and the stress for the amorphous-to-Zr_2_Ni transformation predicted by Eq. (2). The calculated energy barriers for nucleation in the compressive and tensile regions are also provided based on the simulation results, agreeing well with the theoretically predicted values.

The relation between *∆*G^*^ and *P* predicted by Eq. (2) is illustrated in Fig. 4. The average tensile and compressive stresses in a shear band are simulated to be approximately + 1 × 10^8 ^Pa and −1.41 × 10^9 ^Pa (Fig. 2c,d), respectively. So, the energy barriers for nucleation in the tensile and compressive regions are calculated to be approximately 1310 kJ/mol and 93.8 kJ/mol, respectively. They are in good agreement with the predicted values of Eq. (2) (Fig. 4), which further verifies application of Eq. (2) for understanding the relation between the energy barriers for nucleation and the loaded stresses.

According to classical nucleation theory, steady-state nucleation rate *I*, i.e., the number of supercritical nuclei formed per unit time in a unit volume of a supercooled liquid, can be expressed as


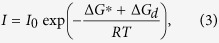


where the pre-exponential term *I*_0_ depends weakly with temperature and varies between 10^41^ and 10^43^ (m^−3^s^−1^)^36^, *R* is the ideal gas constant. Since the viscosity in individual shear bands is similar to that in the supercooled liquid state^21^, the temperature *T* for calculation of the kinetic contribution to the nucleation rate is selected to be 700 K, which is slightly higher than the glass transition temperature of 696 K^14^. It’s worth explaining that the selection of the temperature of 700 K does not mean a considerable temperature increase in shear bands, but is based on the consideration that the viscosity is in a similar range of the supercooled liquid state. The room temperature of 300 K is selected for evaluation of the thermodynamic contribution to nucleation rate. Using the value of ∆G^*^ (Fig. 4), the nucleation rate under the compressive stress of 1.41 × 10^9 ^Pa is estimated to be approximately 5.12 × 10^16 ^m^−3^s^−1^ according to Eq. (3). However, the nucleation rate approaches 0 under the tensile stress of 1 × 10^8 ^Pa, which is the reason why no nanocrystals are observed in the tensile centre regions of shear bands (Fig. 1).

In summary, we directly observe that nanocrystals precipitate in shear bands of Zr_60_Al_15_Ni_25_ metallic glass subjected to cold rolling and find that the nanocrystallization preferentially occurs in the compressive regions of individual shear bands neighboring the undeformed amorphous matrix. The localized nanocrystallization in individual shear bands is attributed to the variation of stresses along the width of a shear band, which is demonstrated by our finite element simulations. The energy barrier for nucleation considerably decreases due to the hydrostatic compressive stress effect. However, the tensile stress effect results in introduction of more excess free volume, which depresses the nucleation. The present work reduces the role of stress state in mechanically induced crystallization in a metallic glass, which convincingly explains the phenomenon that the nanocrystallization only occurs on the compressive side of a bent metallic glass^13^.

## Methods

Specimens with a nominal composition of Zr_60_Al_15_Ni_25_ (in atomic percent) were prepared by suction casting in a copper mold under an argon atmosphere. The amorphous nature of the as-cast specimens is verified by X-ray diffraction (XRD) using monochromatic Co-K_α_ radiation and transmission electron microscopy (TEM). The as-cast bulk specimens were cut into bars with a cross-section of 1 mm × 2 mm and a length of 10 mm for cold rolling. The strain rates were controlled to be 10^−3^ ~ 10^−4^ s^−1^. The deformation degree was evaluated by the reduction in thickness, i.e., *ε* = (*h*_0_ − *h*)/*h*_0_, where *h*_0_ and *h* were the sample thickness before and after rolling, respectively. The microstructures of shear bands were observed by high-resolution transmission electron microscopy (HRTEM) under an accelerating voltage of 200 kV, equipped with a Gatan Image Filter System (GIF 2000) and an energy dispersive X-ray spectrometer (EDS, Oxford). The TEM foils with a diameter of 3 mm were thinned using a twin-jet thinning electropolisher in a solution of 5% (volume percent) perchloric acid and 95% ethanol at 243 K. The chemical compositions of amorphous matrix and shear bands were checked by EDS and electron energy loss spectroscopy (EELS).

The finite element simulations were performed by specialized ANSYS software. A stress-strain curve of Zr_60_Al_15_Ni_25_ bulk metallic glass (see Supplementary Information, Fig. S5), obtained by uniaxial compression testing at a strain rate of 10^−4^ s^−1^, was used for the constitutive law of simulations. Its yield stress and elastic modulus are approximately 1.8 × 10^9 ^Pa and 8.6 × 10^10 ^Pa, respectively. A Poisson ratio *ν* of 0.38 was selected for rolling simulation^37^. The simulation of rolling was modeled and shown in Fig. 2a. The samples were rolled continuously along one direction to obtain the desired deformation degrees. For the simulations, the rolled samples have a cross-section of 1 mm (height) × 10 mm (width) and a length of 25 mm. The rollers were defined as rigid materials. Mesh refinement was used to guarantee the simulation accuracy.

## Additional Information

**How to cite this article**: Yan, Z. *et al.* Localized crystallization in shear bands of a metallic glass. *Sci. Rep.* **6**, 19358; doi: 10.1038/srep19358 (2016).

## Supplementary Material

Supplementary Information

## Figures and Tables

**Table 1:** Calculated thermodynamic data used for evaluation of ΔG^*^.

*V*_m_^c^ (m^3^/mol)	*V*_m_^a^ (m^3^/mol)	*E*_e_ (J/mol)	Δ*G*_m_ (J/mol)
1.34 × 10^−4^	1.55 × 10^−4^	15580	−1719
