# Supplementary material for: Localized crystallization in shear bands of a metallic glass
Source: Sci Rep. 2016 Jan 13;6:19358. doi: 10.1038/srep19358 (PMC4725363; doi:10.1038/srep19358)
Supplement: Supplementary Information [file srep19358-s1.doc]

**Supplementary Information for**

**Localized crystallization in shear bands of a metallic glass**

Zhijie Yan^1,2*^, Kaikai Song^2,3^, Yong Hu^1*^, Fuping Dai^4^, Zhibing Chu^1^ & Jürgen Eckert^5,6^

^1^School of Materials Science and Engineering, Taiyuan University of Science and Technology,

Taiyuan, 030024, P. R. China

^2^IFW Dresden, Institut für Komplexe Materialien, Helmholtzstraße 20, D-01069 Dresden, Germany

^3^School of Mechanical, Electrical & Information Engineering, Shandong University (Weihai), Weihai 264209, P.R. China.

^4^Northwestern Polytechnical University, Xi’an 710072, P.R. China

^5^ Erich Schmid Institute of Materials Science, Austrian Academy of Sciences, Jahnstraße 12, A-8700 Leoben, Austria

^6^ Department of Materials Physics, Montanuniversität Leoben, Jahnstraße 12, A-8700 Leoben, Austria

^*^Correspondence and requests for materials should be addressed to Z.Y. (zjyan@tyust.edu.cn) or Y.H. (hytyust@163.com)

Supplementary Information including:

Figures S1 to S6


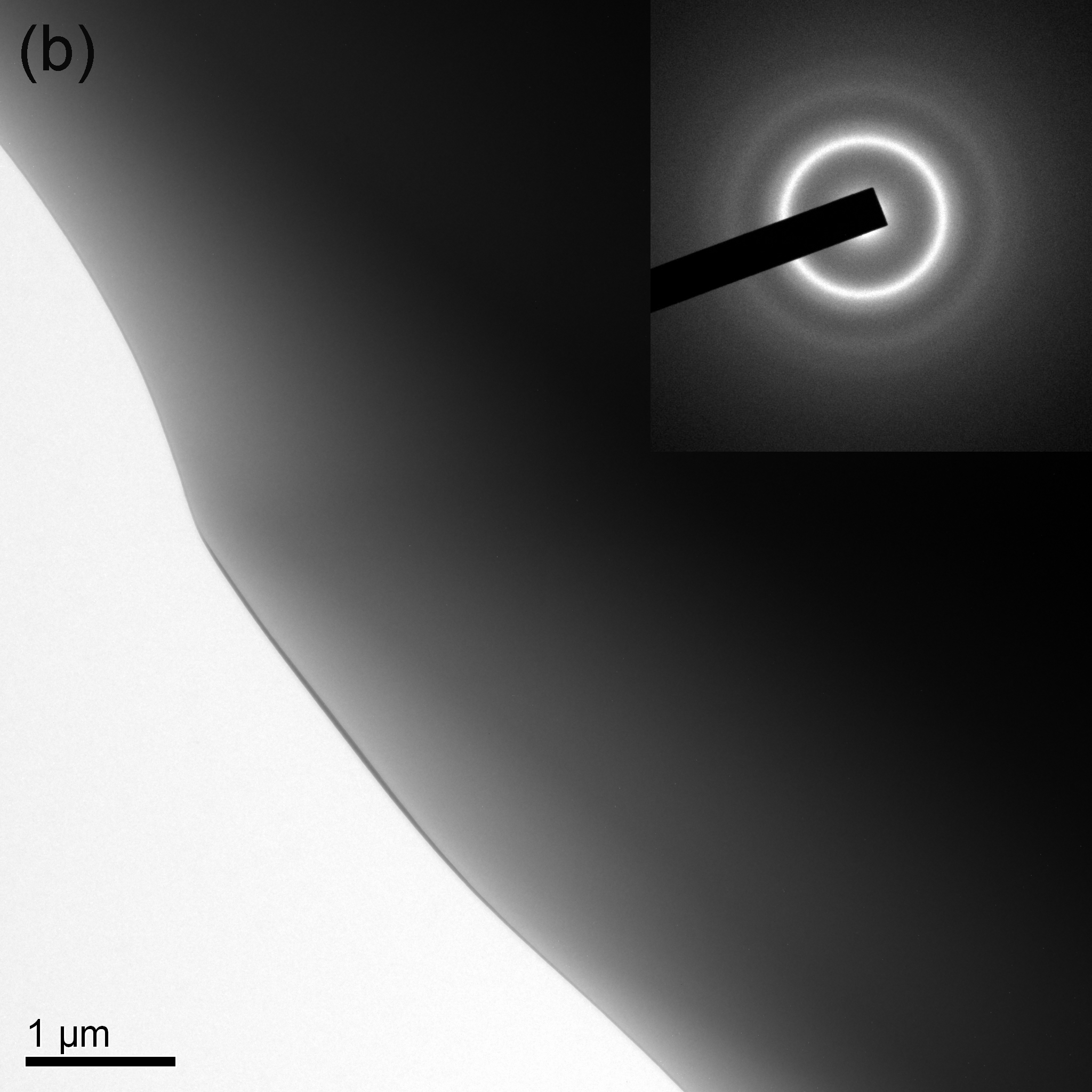


Fig. S1 XRD pattern (a) and TEM image (b) of an as-cast Zr_60_Al_15_Ni_25_ bulk specimen, verifying its amorphous nature.


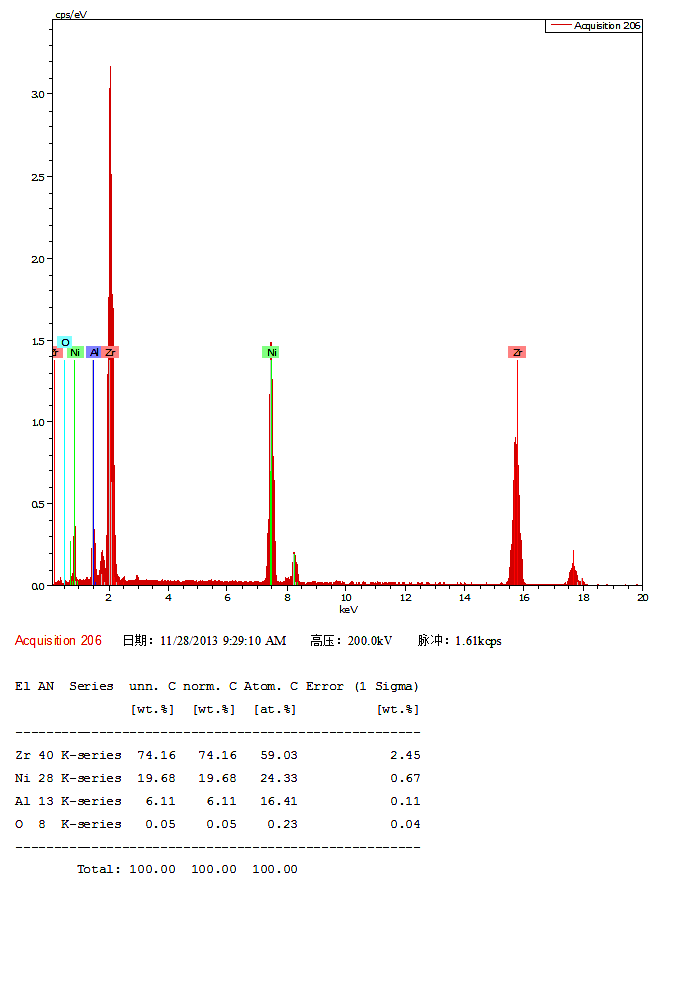


Fig. S2 EDS pattern of the amorphous matrix, indicating that the average chemical composition is Zr_59.03_Al_24.33_Ni_16.41_, being consistent with the nominal composition in addition to a negligible content of oxygen.


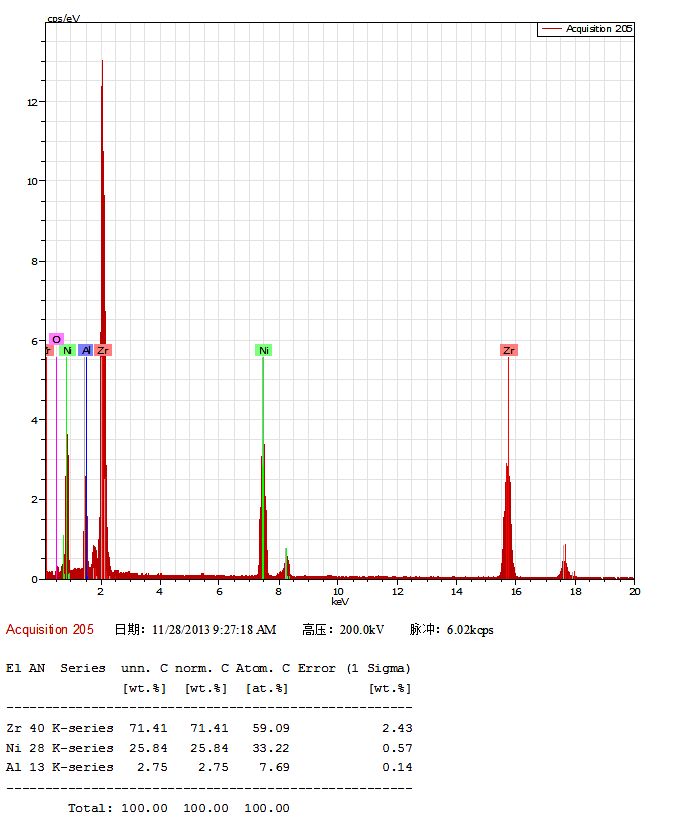


Fig. S3 EDS pattern from a shear band, indicating that its average chemical composition is Zr_59.09_Al_33.22_Ni_7.69_, suggesting considerable redistribution of atoms within individual shear bands due to cold rolling.


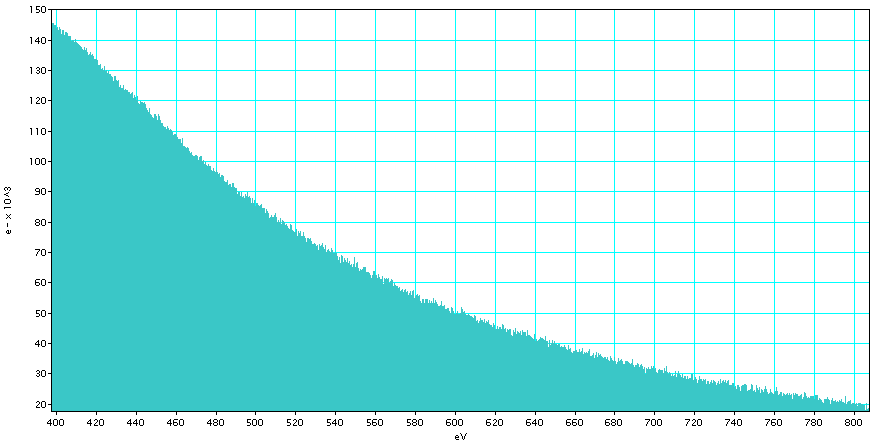


Fig. S4 EELS pattern of oxygen from a shear band, verifying that the oxygen content is negligible within shear bands.


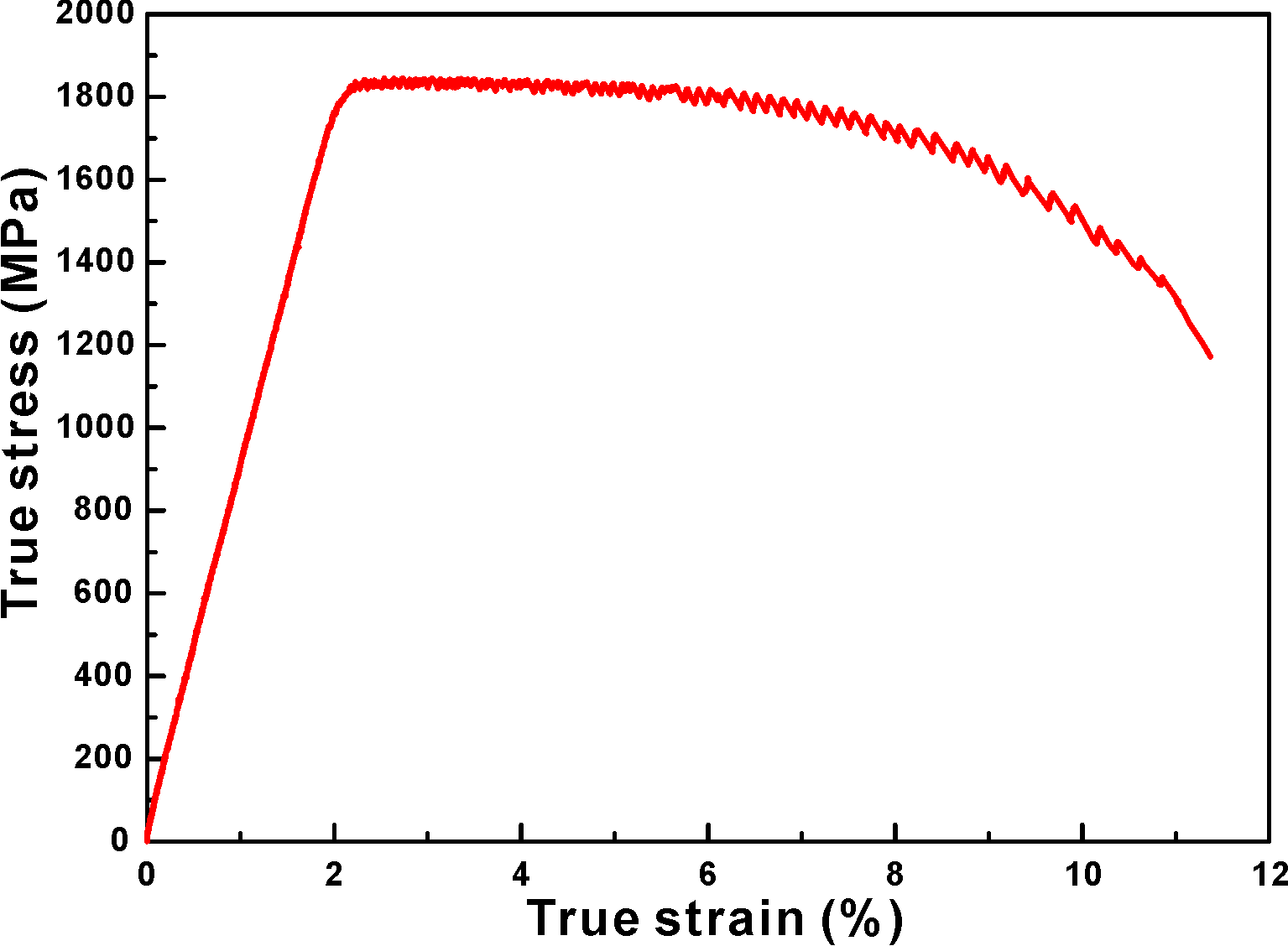


Fig. S5 Uniaxial compression stress-strain curve of Zr_60_Al_15_Ni_25_ bulk metallic glass at a strain rate of 10^-4^ s^-1^.


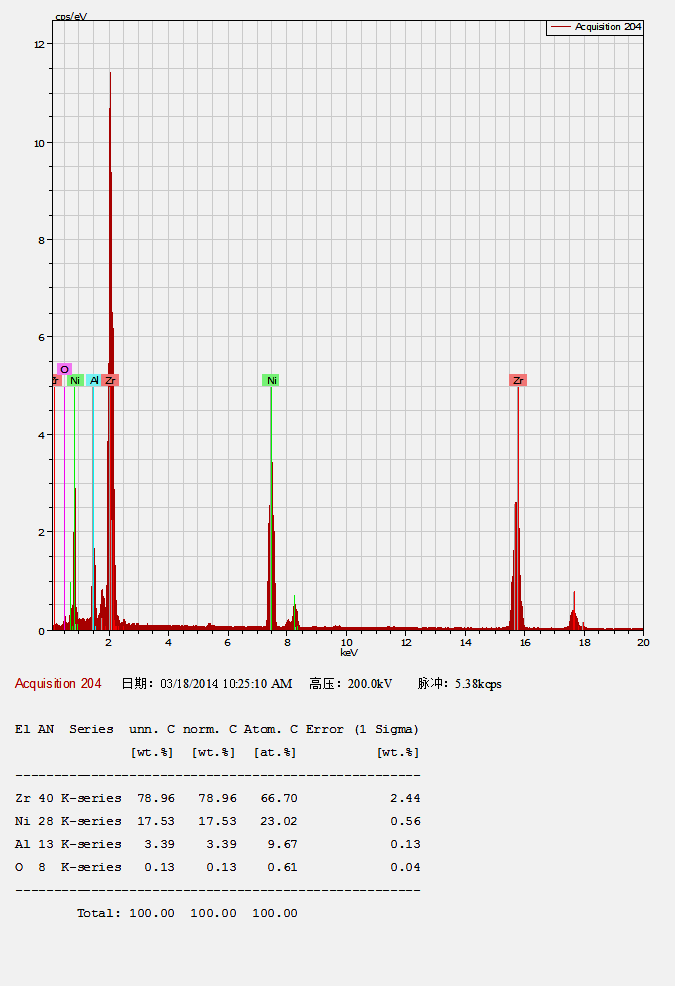


Fig. S6 EDS pattern from a precipitated phase, indicating that its composition is consistent with the stoichiometric concentration of Zr_2_(AlNi).
